# A regulatory circuit between Kaposi sarcoma-associated herpesvirus and host innate immune system

**DOI:** 10.1186/1750-9378-7-S1-P28

**Published:** 2012-04-19

**Authors:** Luwen Zhang

**Affiliations:** 1 School of Biological Sciences, Nebraska Center for Virology, University of Nebraska, Lincoln, NE, USA

## 

Kaposi sarcoma-associated herpesvirus (KSHV) is a human γ-herpesvirus associated with several human malignancies. The replication and transcription activator (RTA) is necessary and sufficient for the switch from KSHV latency to lytic replication. Toll-interleukin-1 receptor (TIR) domain-containing adaptor-inducing β-interferon (TRIF, also called TIR-domain-containing adaptor molecule-1 (TICAM-1)) is a signaling adaptor molecule that is critically involved in the Toll-like receptor 3 (TLR-3) and TLR-4 signaling pathways for type I interferon (IFN) production, a key component of innate immunity against microbial infection. Previously we have identified that RTA blocks TLR3 signaling activation by degrading cellular TRIF, and this RTA-mediated degradation is at least partially mediated through the ubiquitin-proteasome pathway. In this report, we have identified a new mechanism that innate immunity regulates KSHV replication. We find that TRIF increases the expression of KSHV RTA. The enhancement of RTA expression and the degradation of TRIF are two independent pathways. TRIF specifically enhances the translation efficiency of RTA mRNA. Because RTA may not directly interact with TRIF, the functional interactions between TRIF and RTA may be indirect through unknown mediators.Taken together, these data suggest that KSHV employs a novel mechanism to block the innate immunity by degrading TRIF protein, and at the same time, use the innate immune system to boost viral replication by increasing the expression of KSHV RTA. This regulatory circuit may be an important part of the KSHV-host interactions for the initial infections. This work may contribute to our understandings on how KSHV interacts with the host immune system for its survival in vivo.

